# Significant Mortality Reduction from Severe *Pneumocystis jirovecii* Pneumonia in People Living with HIV and Treated in the Intensive Care Unit, Croatia, 2002–2023

**DOI:** 10.3390/pathogens14100973

**Published:** 2025-09-25

**Authors:** Filip Glavač, Lucija Dragošević, Josip Begovac, Marko Kutleša, Anita Atelj, Martina Vargović, Šime Zekan, Đivo Ljubičić, Ana Čičmak, Marija Santini

**Affiliations:** 1 Department for Infections in Immunocompromised, University Hospital for Infectious Diseases Zagreb, 10000 Zagreb, Croatia; 2 School of Medicine, University of Zagreb, 10000 Zagreb, Croatia; 3 Dubrava University Hospital, 10000 Zagreb, Croatia; 4 Department for Mycology, Croatian Institute of Public Health, 10000 Zagreb, Croatia

**Keywords:** *Pneumocystis jirovecii*, pneumonia, HIV, acquired immunodeficiency syndrome, intensive care units, invasive mechanical ventilation, extracorporeal membrane oxygenation

## Abstract

*Pneumocystis jirovecii* pneumonia (PCP) remains a frequent cause of intensive care unit (ICU) admission among people living with HIV (PLWH), despite widespread antiretroviral therapy (ART) use. We conducted a retrospective cohort study of 39 PLWH with PCP admitted to the ICU at the Croatian national HIV referral center between 2002 and 2023. Patients were grouped by calendar period (pre-2015 vs. post-2015, reflecting the adoption of the “test and treat” strategy in 2015). Primary outcomes included ICU, 30-day, and 1-year mortality. We also evaluated the association between in-ICU ART initiation and survival. There were 37 (94.9%) males with a median age of 49 years (Q1–Q3, 37.5–54.5). Thirty-three (84.6%) were newly diagnosed with HIV. There were no differences between the observed periods regarding demographic characteristics. ART was initiated in the ICU in 21 (53.8%) patients, more frequently after 2015 (*p* < 0.001). ICU, 30-day, and 1-year mortality rates were 53.9% (*n* = 21), 51.3% (*n* = 20), and 66.7% (*n* = 26), respectively. Survival significantly improved in the later period, with 1-year survival reaching 54.5% (12/22). In-ICU ART initiation was associated with improved survival in univariable analysis, but this effect attenuated after adjusting for APACHE II or calendar year. Early ART may offer benefit but remains confounded by disease severity and evolving care standards.

## 1. Introduction

*Pneumocystis jirovecii* is an unusual microorganism that was initially thought to be a protozoan. However, ribosomal RNA analysis has shown that it is homologous to fungi [1,2]. Standard microbiological methods cannot cultivate it, it does not contain ergosterol in its cell wall, and it is treated with antibiotics. The primary reservoir for *Pneumocystis* remains unidentified. Its spread is airborne [3,4]. Infection can manifest clinically either through the reactivation of a previously acquired pathogen [5] or through its de novo acquisition [6]. *Pneumocystis jirovecii* has demonstrated its pathogenic potential since the mid-twentieth century, primarily causing pneumonia in premature and malnourished children [7]. It became particularly prominent in the 1970s and early 1980s as a significant cause of pneumonia in patients with the newly defined acquired immunodeficiency syndrome (AIDS), which was later identified to be caused by HIV. *Pneumocystis* pneumonia (PCP) was the first AIDS-defining opportunistic infection accepted by the U.S. Centers for Disease Control and Prevention (CDC) [8].

The incidence of PCP has declined significantly with the introduction of antiretroviral therapy (ART) and primary prophylaxis. For instance, in the United States, the incidence of PCP among people living with HIV (PLWH) was 3.0 cases per 100 person-years between 1994 and 1997, dropping to 0.39 cases per 100 person-years from 2008 to 2010 [9,10]. In Uganda, the rate of bronchoscopies conducted for suspected PCP in hospitalized PLWH decreased from 40% from 1999 to 2000 to just 10% from 2007 to 2008 [11,12]. Similarly, in Spain, the incidence of PCP fell from 1.3 cases per 100 person-years in 2000 to 0.3 cases per 100 person-years by 2013 [13].

Despite being preventable and declining in incidence rates, PCP remains a leading AIDS-defining opportunistic infection, often requiring intensive care treatment [14]. The highest risk is for PLWH presenting late, with CD4+ counts below 200/µL, who are unaware of their HIV status, or with difficulty adhering to ART. PCP is associated with substantial mortality rates varying between 15 and 32% depending on different regions [15,16], but this data does not reflect the mortality of critically ill patients. Some studies indicate that the mortality rate for the most severely ill patients receiving complex intensive care measures remains above 50% [17]. PCP continues to be an important cause of ICU admission for PLWH [18], and mechanical ventilation is often required in these patients, so mortality in this setting remains high. The benefits of ART being started before or during ICU admission for PCP-related critical illness remain uncertain.

We conducted a retrospective cohort study of PLWH with PCP requiring intensive care at a national HIV referral center in Croatia over a 22-year period. Our aim was to examine ICU, 30-day, and 1-year mortality, assess whether outcomes improved over time (before and after 2015 when the “test and treat” ART strategy became widely accepted), and evaluate whether the initiation of ART before or during the ICU stay was associated with survival.

## 2. Materials and Methods

### 2.1. Setting

This retrospective observational cohort study was conducted at the University Hospital for Infectious Diseases (UHID) in Zagreb, Croatia, from 1 January 2002 to 31 December 2023. The study is reported in accordance with the Strengthening the Reporting of Observational Studies in Epidemiology (STROBE) guidelines [19]. UHID is a 200-bed tertiary teaching hospital and the national Reference Centre for HIV. In Croatia, care for PLWH is centralized, and all patients are monitored and receive ART at UHID. As of 2024, more than 2000 PLWH received care at UHID. Croatia has a low incidence of HIV infection, with 2.3 new cases per 100,000 population (compared to an EU/EEA average of 5.3 per 100,000 in 2023) [20]. The Department for Intensive Care Medicine at UHID is an 18-bed ICU specialized in infectious diseases and serves as the national Reference Center for veno-venous extracorporeal membrane oxygenation (VV ECMO).

### 2.2. Population and Definitions

We retrospectively reviewed paper-based and electronic medical records of all PLWH aged 18 years or older who had a diagnosis of PCP and required ICU care for more than 24 h between 2002 and 2023. This included individuals admitted directly to the ICU from UHID’s Emergency Outpatient Department, transferred from other inpatient departments at UHID, or referred from external institutions. There were no formal protocols for ICU admission during the study period; decisions regarding ICU admission, PCP diagnosis and treatment, and ART initiation were made collaboratively by ICU physicians and HIV specialists. Immune reconstitution inflammatory syndrome (IRIS) was defined according to the 2009 AIDS Clinical Trials Group (ACTG) criteria.

### 2.3. Variables

We collected the following data: age at ICU admission, sex assigned at birth, mode of HIV acquisition, Charlson comorbidity index, Acute Physiology and Chronic Health Evaluation II (APACHE II) score, diagnosis of *Pneumocystis jirovecii* infection, ICU treatments received (invasive mechanical ventilation, VV ECMO, continuous renal replacement therapy), and mortality. The primary outcomes were ICU mortality, 30-day mortality, and 12-month mortality following ICU admission.

### 2.4. Statistical Analysis

We present our categorical data by frequencies with percentages and continuous data by medians with first (Q1) and third (Q3) quartiles. Continuous variables were compared between the two study periods (2002–2015 vs. 2016–2023) using the Mann–Whitney test, while categorical variables were analyzed using the chi-squared test or Fisher’s exact test with mid-p adjustment. Survival was analyzed using the Kaplan–Meier method, with differences between groups being assessed using the log-rank test.

To evaluate the association between ART exposure and mortality, univariable and limited multivariable regression models were used. For 30-day and 1-year mortality analyses, ART exposure was defined based on initiation prior to or during the ICU stay. Individuals who initiated ART after ICU discharge were classified as unexposed. This definition was chosen to reflect the impact of early ART initiation and to avoid immortal time and survivor biases associated with post-ICU discharge treatment initiation.

ICU mortality was analyzed using logistic regression, and 30-day and 1-year mortality were analyzed using Cox proportional hazards models. Due to the limited number of events (21 ICU deaths, 20 deaths at 30 days, and 26 deaths at 1 year), multivariable models were restricted to a maximum of two predictors to reduce the risk of overfitting. ART exposure was included as the main variable of interest. Calendar period (2016–2023 vs. 2002–2015) and APACHE II score (continuous) were evaluated as potential covariates. Because ART use was strongly associated with calendar period (*p* < 0.001), we examined the potential for collinearity. Variance inflation factors (VIFs) were 1.7 for ART and 1.6 for calendar period, indicating low collinearity. The proportional hazards assumption for Cox models was tested using Schoenfeld residuals. As a sensitivity analysis, we repeated all univariable analyses after excluding patients without microbiologically documented PCP. The results are presented in the Supplementary Materials. All statistical analyses were performed using SAS version 9.4 (SAS Institute, Cary, NC, USA), with a two-sided significance threshold of *p* < 0.05.

## 3. Results

### 3.1. Demographic and Clinical Characteristics

A total of 1979 adult PLWH were treated at UHID between 2002 and 2023. Of these, 450 (22.7%) were treated for an AIDS-defining illness. Among the 175 who had PCP, 39 (22.3%) required ICU treatment. PLWH with PCP were admitted to the ICU either from other hospitals (*n* = 33) or after being admitted and treated at the Department for Immunocompromised Patients at UHID (*n* = 6).

PLWH treated in the ICU were mainly male (*n* = 38, 95.9%), the median age was 49.0 years (Q1–Q3, 37.5–54.5 years), and the main mode of HIV acquisition was sex between men (*n* = 27, 64.5%). The main demographic and clinical characteristics according to study periods are presented in Table 1. The diagnosis of PCP was most commonly made by directly identifying *Pneumocystis jirovecii* in bronchoalveolar lavage (BAL) fluid using Giemsa staining or through a positive PCR test from BAL samples. However, the majority of cases that were diagnosed on clinical grounds were from the period of 2002 to 2015 (9 of 12, Table 1).

The median APACHE II score was somewhat higher during the period of 2002–2015 compared to 2015–2023, and PLWH from 2002 to 2015 more frequently had a high APACHE II score, although these differences were not statistically significant (Table 1). The severity of PCP was reflected in the high number of PLWH requiring invasive mechanical ventilation (32 of 39, 82.1%); six (15.4%) received veno-venous extracorporeal membrane oxygenation (ECMO), and seven (17.9%) required continuous renal replacement therapy (Table 1). The median duration of veno-venous extracorporeal membrane oxygenation (VV ECMO) was 10.0 days (Q1–Q3, 7.0–13.0). The median duration of continuous renal replacement therapy (CRRT) was 3.5 days (Q1–Q3, 2.0–6.0).

### 3.2. Antiretroviral Therapy

A total of 21 (53.8%) of 39 individuals received ART during the ICU stay, which was initiated before ICU admission in 5 individuals and during the ICU stay in 16. The remaining 18 patients were not on ART during ICU treatment; among them, 4 initiated ART after ICU discharge. Among those who started ART before ICU admission, the median time from ART initiation to ICU admission was 6 days (range: 2 to 19). One patient was admitted to the ICU due to suspected unmasking PCP-associated immune reconstitution inflammatory syndrome (IRIS). Among those who initiated ART during their ICU stay, the median time to ART initiation was 12.5 days after ICU admission (range: 1 to 27). ART was administered more frequently during the period of 2016–2023 (17 of 22 patients) compared to 2002–2015 (4 of 17 patients; *p* < 0.001). Individuals who received ART in the ICU had a lower APACHE II score (median: 16.0; Q1–Q3: 14.0–21.0) compared to those who did not receive ART (median: 22.5; Q1–Q3: 14.0–28.0), although this difference was not statistically significant (*p* = 0.085).

### 3.3. Mortality Outcomes

**Figure 1:**
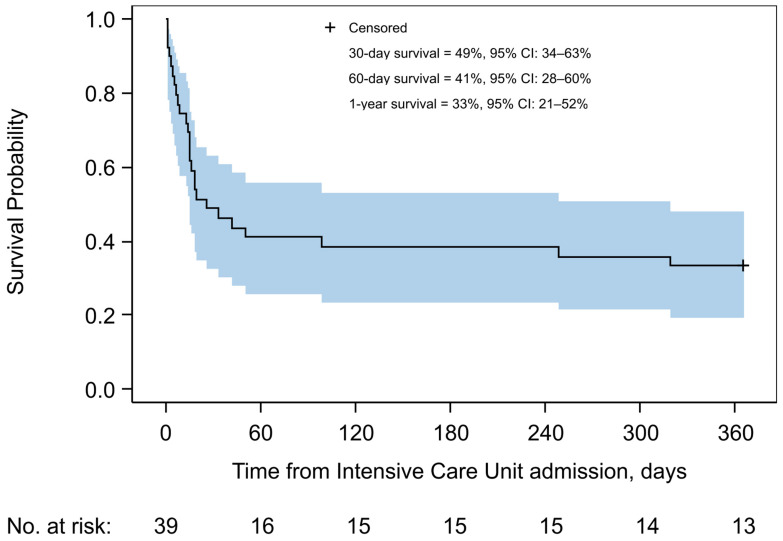
Kaplan–Meier estimate of overall survival in the study cohort. Shown is the cumulative probability of survival up to 1 year after intensive care unit admission for people with HIV with *Pneumocystis* pneumonia, from 2002 to 2023. The solid line represents the Kaplan–Meier survival estimate, and the shaded area indicates the 95% confidence interval. The estimated probabilities of survival at 30 days, 60 days, and 1 year are shown inside the graph. The number of individuals at risk is shown below the curve.

Twenty-one PLWH (53.9%) died during the ICU stay. The 30-day and 1-year mortality rates were 51.3% (*n* = 20) and 66.7% (*n* = 26), respectively. Most deaths occurred within the first 60 days of follow-up; the estimated probability of survival at day 60 was 0.41 (95% confidence interval [CI]: 0.28 to 0.60) (Figure 1). The median time to death was 26 days, and the estimated probability of survival at 1 year was 0.33 (95% CI: 0.21 to 0.52).

Both a more recent calendar period and lower APACHE II scores were significantly associated with improved survival during ICU stay, as well as at 30 days and 1 year after ICU admission (Table 1 and Table 2, Supplementary Figure S1). The estimated 30-day survival was 68% in the 2016–2023 period, compared to 24% in 2002–2015 (Supplementary Figure S1, Panel A). Among individuals who required invasive mechanical ventilation (*n* = 32), ICU mortality was 65.6% (*n* = 21). This was significantly higher in the earlier period: 87.5% (14 of 16) in 2002–2015, compared to 43.8% (7 of 16) in 2016–2023 (*p* = 0.013). Of the six individuals who received veno-venous extracorporeal membrane oxygenation (VV ECMO), three died during their ICU stay. Among the 11 individuals treated with non-invasive mechanical ventilation (NIMV), 3 (27.3%) died during ICU treatment. Of the six individuals who developed IRIS, three died during their ICU stay.

Regression analyses suggested a potential benefit of early ART initiation on ICU, 30-day, and 1-year mortality; however, these associations were attenuated after adjustment for APACHE II score or calendar period (Table 2). Kaplan–Meier survival curves supported these findings, showing significantly better 30-day survival among patients who received ART before or during the ICU stay (*p* = 0.014) (Supplementary Figure S1, Panel B). At 1 year, the survival difference based on ART exposure approached statistical significance (*p* = 0.06) (Supplementary Figure S1, Panel D).

## 4. Discussion

We conducted a retrospective observational study of severe PCP in PLWH, comparing clinical characteristics and outcomes before and after the implementation of the “test and treat” approach to antiretroviral therapy (ART) in 2015 [21]. Despite notable improvements, overall mortality remained high, with more than half of patients dying during their ICU stay and approximately two-thirds within one year. However, survival significantly improved among those treated during the 2016–2023 period, suggesting progress in both HIV care and intensive care management. Notably, one-year survival reached 55% in this more recent period (Supplementary Figure S1, Panel C).

Other studies have similarly reported high mortality in ICU-treated PLWH with PCP. For example, Chiliza et al. (2018) reported a 90-day mortality rate of 61.9% [22]; Schmidt et al. (2018) reported 58% [23]; Giacobbe (2023) found 30- and 90-day mortality rates of 52% and 67%, respectively [14]; Wang et al. (2024) reported 70% 28-day mortality [24]; and Kamel et al. (2024) reported an in-hospital mortality of 28.5% [18]. The variability in these outcomes likely reflects differences in study populations and ICU admission criteria.

Our findings suggest a potential benefit of initiating ART during or shortly before ICU admission. In univariable analyses, ART use was associated with lower ICU and 30-day mortality, though these associations weakened after adjusting for calendar period or disease severity (APACHE II score). ART use was strongly correlated with the more recent treatment period (*p* < 0.001). Although the VIF values for ART use and calendar period were low (<2), suggesting that collinearity was not a major statistical concern, the strong temporal overlap between these variables complicates the interpretation of their independent effects. This supports the idea that improved survival may result from a combination of factors, including overall improvements in HIV and critical care and more frequent ART use.

The benefit of early ART initiation in patients with PCP requiring intensive care remains uncertain. Although early ART has been associated with reduced mortality in the general population of individuals with opportunistic infections [25], its role in critically ill patients is less clear. A randomized trial from Brazil, which did not reach its planned sample size, found no survival benefit from early ART initiation among critically ill PLWH admitted to the ICU. Of the 115 participants enrolled, all required mechanical ventilation and 60 had PCP; the overall ICU mortality was 47.8% [26]. Observational studies have likewise not demonstrated a consistent independent benefit of early ART in ICU patients with PCP [27,28]. Our findings reflect this complex clinical context: while early ART may confer some benefit, its specific contribution is difficult to disentangle from concurrent improvements in HIV care and intensive care practices.

Initiating ART in the ICU presents practical challenges [29]. These include uncertainty regarding drug absorption via nasogastric tube, dose adjustments in renal failure (including during continuous renal replacement therapy or VV ECMO), and the risk of IRIS. However, newer ART regimens with simpler dosing and better safety profiles help mitigate these issues. In our cohort, the increased use of ART in the ICU likely reflects broader improvements in the management of PLWH in critical care settings.

A high proportion of our patients (82.1%) required invasive mechanical ventilation, which was strongly associated with poor outcomes. Among invasively ventilated patients, ICU mortality was 65.6%, although this improved markedly over time—from 87.5% in 2002–2015 to 43.8% in 2016–2023. This suggests substantial progress in the management of severe PCP, likely driven by earlier ICU admission, advances in invasive mechanical ventilation strategies, and greater clinical experience. Historically, ICU mortality in this group exceeded 80%, and some cohorts reported rates approaching 100% [28,30]. A UK study (1990–2005) reported a survival rate of only 35.3% among 34 ventilated patients with HIV-associated PCP [27].

The use of non-invasive mechanical ventilation (NIMV) increased during the more recent period and should be considered more often, as it may prevent the need for intubation and its consequences in many PLWH with PCP-related respiratory failure [31].

VV ECMO was used in six patients, with three surviving. This modality has been available at our institution (UHID ICU) for treating acute respiratory distress syndrome since 2009 and for severe PCP since 2012. It is noteworthy that when first introduced, severe PCP was considered a relative contraindication for ECMO.

Suspected IRIS occurred in 6 of 21 (28.6%) PLWH who were on ART, a higher proportion than reported in other studies, where rates ranged from 5% to 12.4% [32,33,34]. Pneumothorax was observed in 22.5% of patients, with no difference between time periods. This rate is higher than the 12.6% recently reported by Kamel et al. [35].

This study has several limitations. First, the small sample size and limited number of outcome events reduced the power of multivariable models, especially for ICU and 30-day mortality. Thus, residual confounding cannot be ruled out. Second, the single-center and observational design limits generalizability and precludes causal inference. Third, we did not include ART initiated after ICU discharge to avoid immortal time bias, but this may have led to underestimation of the long-term impact of ART. Fourth, using calendar period as a proxy for improvements in care may not fully capture specific clinical advances over time. Furthermore, several of the confidence intervals were wide, indicating limited statistical power and cautioning against overinterpretation of point estimates. This imprecision is likely due to the relatively small sample size and number of outcome events. Finally, in almost one-third of patients, the diagnosis of PCP was based on clinical grounds rather than microbiological confirmation, reflecting the challenges of organizing bronchoscopy in critically ill PLWH at UHID. In a sensitivity analysis restricted to patients with microbiologically documented PCP (*n* = 27), the results showed a similar tendency, but the strengths of the associations were weaker or lost statistical significance due to the reduced sample size (Supplementary Figure S2 and Table S1).

Nevertheless, recent data on ICU treatment of severe PCP are scarce, and to our knowledge, no similar study has been reported from Central or Southeast Europe. Our patient population was severely immunosuppressed, with extremely low median CD4+ T-cell counts and high HIV-1 RNA levels, representing a particularly vulnerable group.

## 5. Conclusions

In this 22-year cohort of PLWH with PCP requiring ICU care, we observed significant improvements in both short- and long-term survival. ART initiation before or during ICU stay was associated with reduced mortality in unadjusted analyses, although this effect diminished after adjusting for disease severity and calendar period. These findings highlight the challenges in isolating the independent effect of ART in critically ill patients and suggest that improved outcomes likely reflect both advances in HIV management and evolving critical care standards. Larger studies are needed to confirm these findings and guide the optimal timing of ART initiation in this high-risk population.

## Figures and Tables

**Table 1:** Basic demographic and clinical characteristics and intensive care features in people living with HIV and treated for *Pneumocystis jirovecii* pneumonia in the Intensive Care Unit, University Hospital for Infectious Diseases, Zagreb, Croatia, 2002–2023.

Characteristics	Period of 2002 to 2015 (*N* = 17)	Period of 2016 to 2023 (*N* = 22)	Total (*N* = 39)	*p*-Value
Age, median (Q1–Q3)	47.0 (38.0–50.0)	50.5 (42.0–58.0)	49.0 (38.0–55.0)	0.160
Male, n (%)	16 (94.1)	21 (95.5)	37 (94.9)	0.748
Mode of HIV acquisition, *n* (%)				
MSM	8 (47.1)	18 (81.8)	26 (66.7)	0.101
Heterosexual	3 (17.6)	1 (4.5)	4 (10.3)	
IDU	1 (5.9)	1 (4.5)	2 (5.1)	
Unknown	5 (29.4)	2 (9.1)	7 (17.9)	
Charlson comorbidity index, median (Q1–Q3)	7.0 (6.0–7.0)	7.0 (6.0–8.0)	7.0 (6.0–8.0)	0.750
APACHE II, median (Q1–Q3)	21.0 (15.0–26.0)	16.0 (13.0–22.0)	20.0 (14.0–25.0)	0.120
APACHE II score categories, *n* (%)				
Low (0 to 10)	0 (0.0)	2 (9.1)	2 (5.1)	0.243
Moderate (11 to 20)	6 (35.3)	12 (54.5)	18 (46.2)	
High (21 to 30)	10 (58.8)	7 (31.8)	17 (43.6)	
Very high (≥31)	1 (5.9)	1 (4.5)	2 (5.1)	
HIV diagnosis ≤ 3 months before admission, *n* (%)	13 (76.5)	20 (90.9)	33 (84.6)	0.370
CD4+ count at admission, cells/μL, median (Q1–Q3)	11.0 (6.0–45.0)	16.5 (5.0–33.0)	14.0 (6.0–44.0)	0.95
HIV-1 RNA (log10 copies/mL), median (Q1–Q3)	5.6 (5.4–5.9)	5.3 (4.9–5.5)	5.4 (5.1–5.7)	0.11
PCP diagnosis, n (%)				0.039
PCR + Cytology ^a^	5 (29.4)	13 (59.1)	18 (46.2)	
PCR only ^a,b^	3 (17.6)	4 (18.2)	7 (17.9)	
Cytology only ^a,c^	0 (0.0)	2 (9.1)	2 (5.1)	
Clinical only	9 (52.9)	3 (13.6)	12 (30.8)	
Patients with multiple opportunistic infections, *n* (%)	3 (17.6)	11 (50.0)	14 (35.9)	0.037
Duration of ICU treatment, days, median (Q1–Q3)	9.0 (2.0–18.0)	17.5 (6.0–27.0)	15.0 (6.0–22.0)	0.109
Non-invasive mechanical ventilation, n (%)	2 (11.8)	9 (40.9)	11 (28.2)	0.053
Invasive mechanical ventilation, *n* (%)	16 (94.1)	16 (72.7)	32 (82.1)	0.110
Invasive mechanical ventilation duration, median (Q1–Q3)	11.5 (5.5–16.5)	18.5 (9.5–32.0)	15.5 (6.5–21.0)	0.070
VV ECMO, *n* (%)	3 (17.6)	3 (13.6)	6 (15.4)	0.840
CRRT, *n* (%)	3 (17.6)	4 (18.2)	7 (17.9)	0.838
Vasopressor support, *n* (%)	9 (52.9)	11 (50.0)	20 (51.3)	0.876
Pneumothorax, *n* (%)	3 (18.8)	5 (22.7)	8 (21.1)	0.855
IRIS, *n* (%)	1 (5.9)	5 (22.7)	6 (15.4)	0.137
Died in ICU, *n* (%)	14 (82.4)	7 (31.8)	21 (53.9)	0.002
In-hospital mortality	15 (88.2)	9 (40.9)	24 (61.5)	0.003
Alive after 30 days ^d^, *n* (%)	4 (23.5)	15 (68.2)	19 (48.7)	0.006
Alive after one year ^d^, *n* (%)	1 (5.9)	12 (54.5)	13 (33.3)	0.001

MSM, men having sex with men; IDU, injection drug use; VV ECMO, veno-venous extracorporeal membrane oxygenation; CRRT, continuous renal replacement treatment; PCP, *Pneumocystis jirovecii* pneumonia; IRIS, immune reconstruction inflammatory syndrome. ^a^ Bronchoalveolar lavage fluid analysis. ^b^ Microscopic examinations were negative. ^c^ PCR was negative in one case and not performed in the other. ^d^ After ICU admission.

**Table 2:** Associations between antiretroviral therapy exposure, study periods, APACHE II score, and mortality in 39 people living with HIV and treated for *Pneumocystis jirovecii* pneumonia in the Intensive Care Unit, University Hospital for Infectious Diseases, Zagreb, Croatia from 2002 to 2023.

Outcome (Regression)	Predictor	Univariable OR or HR (95% CI)	*p*-Value	ART Adjusted for Period, OR or HR (95% CI)	*p*-Value	ART Adjusted for APACHE II, OR or HR (95% CI)	*p*-Value
ICU mortality (logistic)	Period (2002–2015 vs. 2016–2023)	10.0 (2.15–46.46)	0.003	7.75 (1.5–42.85)	0.019	–	
	ART (yes vs. no)	0.24 (0.06–0.92)	0.037	0.60 (0.11–3.10)	0.537	0.32 (0.07–1.49)	0.147
	APACHE II (per point)	1.22 (1.06–1.39)	0.006	–		1.21 (1.04–1.40)	0.012
30-day mortality (Cox)	Period (2002–2015 vs. 2016–2023)	3.38 (1.33–8.44)	0.011	2.39 (0.81–7.06)	0.116	–	
	ART (yes vs. no)	0.33 (0.13–0.84)	0.020	0.53 (0.18–1.56)	0.250	0.47 (0.17–1.23)	0.131
	APACHE II (per point)	1.11 (1.04–1.18)	0.003	–		1.09 (1.01–1.17)	0.022
1-year mortality (Cox)	Period (2002–2015 vs. 2016–2023)	3.80 (1.67–8.62)	0.001	3.64 (1.41–9.40)	0.008	–	
	ART (yes vs. no)	0.49 (0.22–1.06)	0.069	0.92 (0.37–2.28)	0.861	0.66 (0.28–1.52)	0.325
	APACHE II (per point)	1.10 (1.03–1.17)	0.003	–		1.08 (1.02–1.16)	0.015

Note: Adjusted models include only ART and one additional covariate (calendar period or APACHE II score) due to limited number of events. ICU = intensive care unit; ART = antiretroviral therapy; OR = Odds Ratio (for ICU mortality); HR = Hazard Ratio (for 30-day and 1-year mortality); CI = confidence interval; – = not in model.

## Data Availability

Research data are available from the corresponding author upon request.

## Supplementary Materials

The following supporting information can be downloaded at https://www.mdpi.com/article/10.3390/pathogens14100973/s1: Figure S1. Kaplan–Meier survival estimates by calendar period and antiretroviral initiation status. Figure S2. Kaplan–Meier survival estimates by calendar period and antiretroviral initiation status in persons with microbiologically confirmed *Pneumocystis* pneumonia (N = 27). Table S1. Associations between antiretroviral therapy exposure, study periods, APACHE II score and mortality in 27 people living with HIV and microbiologically confirmed *Pneumocystis jirovecii* pneumonia treated in the Intensive Care Unit, University Hospital for Infectious Diseases, Zagreb, Croatia from 2002 to 2023.

## Abbreviations

The following abbreviations are used in this manuscript:

ACTG	AIDS Clinical Trials Group
AIDS	Acquired immunodeficiency syndrome
APACHE II	Acute Physiology and Chronic Health Evaluation II
ART	Antiretroviral therapy
BAL	Bronchoalveolar lavage
CDC	Centers for Disease Control and Prevention
CI	Confidence interval
CRRT	Continuous renal replacement therapy
ECMO	Extracorporeal membrane oxygenation
HIV	Human immunodeficiency virus
ICU	Intensive care unit
IDU	Injection drug use
IRIS	Immune reconstitution inflammatory syndrome
MSM	Men having sex with men
NIMV	Non-invasive mechanical ventilation
PCP	*Pneumocystis jiroveci* pneumonia
PLWH	People living with HIV
Q1	First quartile
Q3	Third quartile
STROBE	Strengthening the Reporting of Observational Studies in Epidemiology
UHID	University Hospital for Infectious Diseases
VV ECMO	Veno-venous extracorporeal membrane oxygenation
