# Supplementary material for: Significant Mortality Reduction from Severe Pneumocystis jirovecii Pneumonia in People Living with HIV and Treated in the Intensive Care Unit, Croatia, 2002–2023
Source: Pathogens. 2025 Sep 25;14(10):973. doi: 10.3390/pathogens14100973 (PMC12567465; doi:10.3390/pathogens14100973)
Supplement: Supplementary file 1 [file pathogens-14-00973-s001.zip › pathogens-3856754-supplementary.pdf]

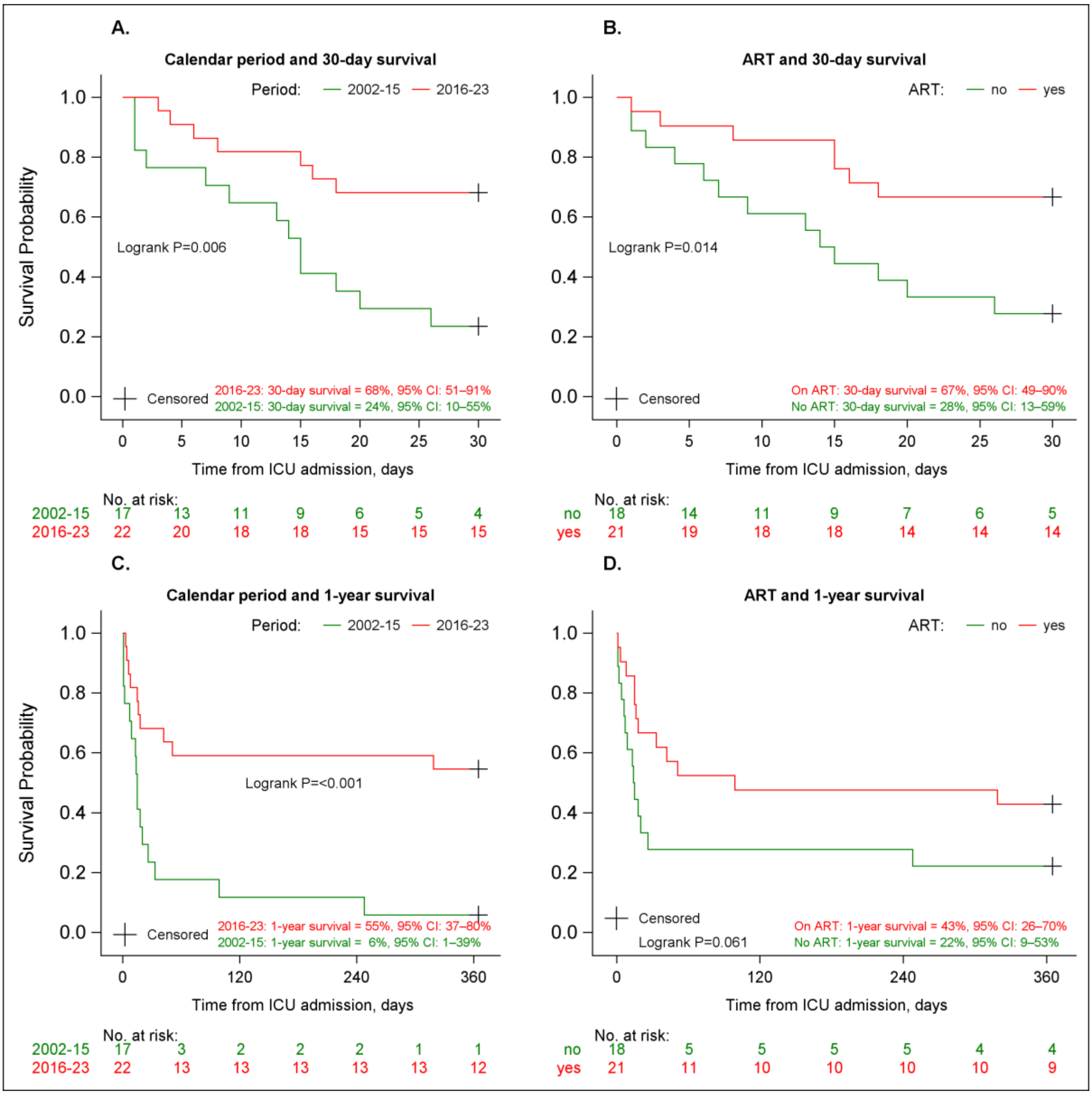

Figure S1. Kaplan–Meier survival estimates by calendar period and antiretroviral initiation status. Panel A shows 30-day survival stratified by calendar period (2002–2015 vs. 2016–2023). Panel B displays 30-day survival according to whether antiretroviral therapy (ART) was initiated before or during the intensive care unit (ICU) stay. Panel C presents 1-year survival by calendar period, and Panel D shows 1-year survival by ART initiation status. In Panels B and D, individuals who initiated ART only after ICU discharge were classified as not receiving ART during the ICU period. Survival differences between groups were assessed using the log-rank test. The number of individuals at risk is shown below each graph.

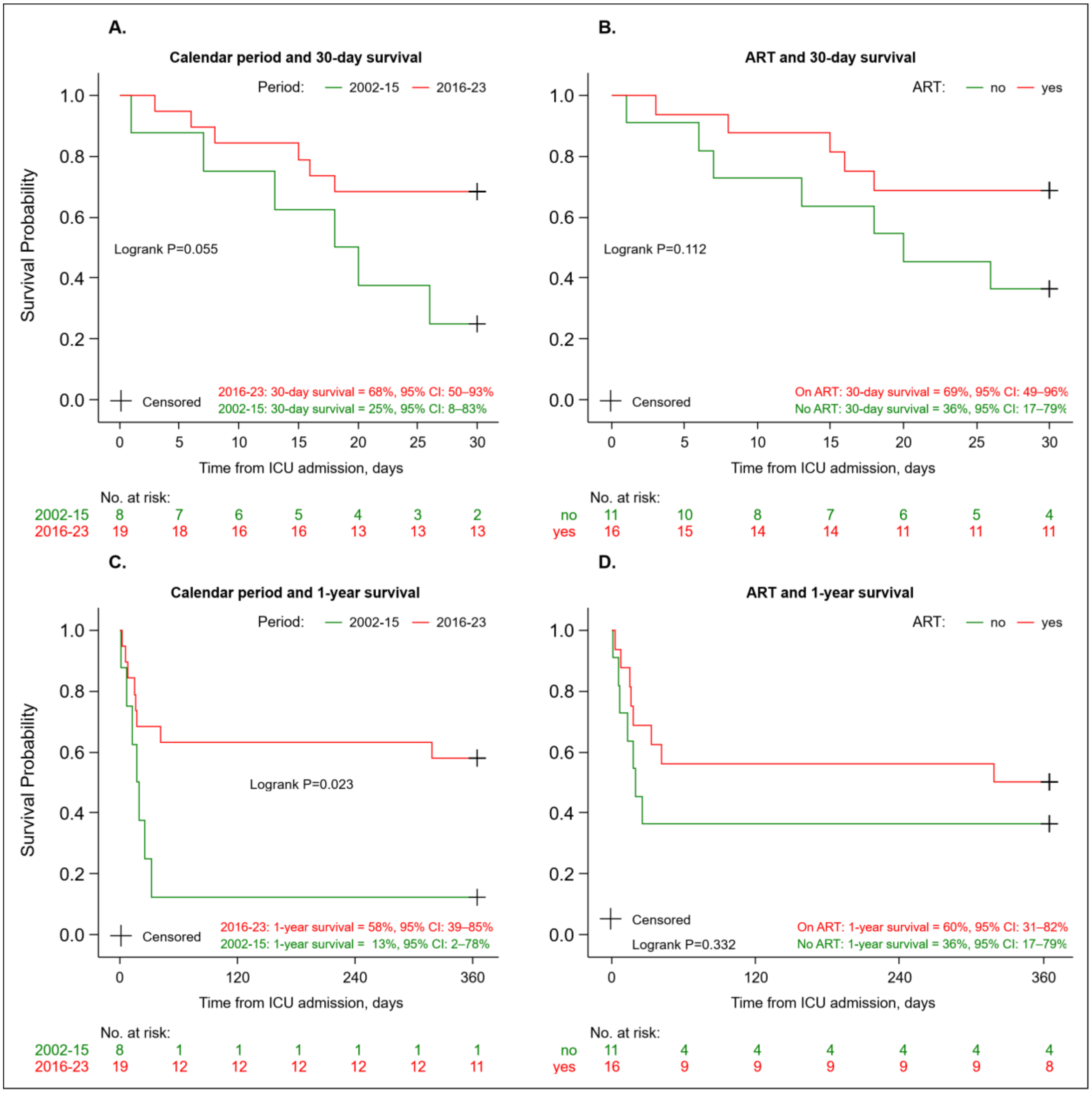

Figure S2. Kaplan–Meier survival estimates by calendar period and antiretroviral initiation status in persons with microbiologically confirmed *Pneumocystis* pneumonia (N=27). Panel A shows 30-day survival stratified by calendar period (2002–2015 vs. 2016–2023). Panel B displays 30-day survival according to whether antiretroviral therapy (ART) was initiated before or during the intensive care unit (ICU) stay. Panel C presents 1-year survival by calendar period, and Panel D shows 1-year survival by ART initiation status. In Panels B and D, individuals who initiated ART only after ICU discharge were classified as not receiving ART during the ICU period. Survival differences between groups were assessed using the log-rank test. The number of individuals at risk is shown below each graph.

Table S1. Associations between antiretroviral therapy exposure, study periods, APACHE II score and mortality in 27 people living with HIV and microbiologically confirmed *Pneumocystis jirovecii* pneumonia treated in the Intensive Care Unit, University Hospital for Infectious Diseases, Zagreb, Croatia from 2002 to 2023.

**Outcome (regression)**	**Predictor**	**Univariable Odds Ratio or Hazard Ratio (95% Confidence Interval)**	***P*- value**
ICU mortality (logistic)	Period (2002–2015 vs. 2016–2023)	15.17 (2.15 – 152.46)	0.021
	ART (yes vs no)	0.34 (0.07 – 1.68)	0.188
	APACHE II (per point)	1.21 (1.02 – 1.42)	0.026
30-day mortality (Cox)	Period (2002–2015 vs. 2016–2023)	2.88 (0.93 – 8-95)	0.068
	ART (yes vs no)	0.41 (0.13 – 1.29)	0.126
	APACHE II (per point)	1.11 (1.00 – 1.24)	0.046
1-year mortality (Cox)	Period (2002–2015 vs. 2016–2023)	3.15 (1.11– 8.96)	0.031
	ART (yes vs no)	0.61 (0.22 – 1.68)	0.339
	APACHE II (per point)	1.10 (1.01 – 1.21)	0.037
